# The Relationship between Cyber-Ostracism and Adolescents’ Non-Suicidal Self-Injury: Mediating Roles of Depression and Experiential Avoidance

**DOI:** 10.3390/ijerph191912236

**Published:** 2022-09-27

**Authors:** Huimin Ding, Liyue Zhu, Hua Wei, Jingyu Geng, Feng Huang, Li Lei

**Affiliations:** 1 School of Education, Renmin University of China, Beijing 100872, China; 2 Zhengzhou Shuqing Medical College, Zhengzhou 450064, China; 3 Normal College, Qingdao University, Qingdao 266071, China; 4 Department of Psychology, Renmin University of China, Beijing 450064, China; 5 Institute of Psychology, Chinese Academy of Sciences, Beijing 100101, China; 6 Department of Psychology, University of Chinese Academy of Sciences, Beijing 100049, China

**Keywords:** cyber-ostracism, non-suicidal self-injury, depression, experiential avoidance

## Abstract

Based on the experiential avoidance model, the current study aims to test the relationship between cyber-ostracism and adolescents’ non-suicidal self-injury and to explore the mediating roles of depression and experiential avoidance. A sample of 1062 middle school students completed questionnaires on cyber-ostracism, depression, experiential avoidance, and self-injurious behavior. The results showed that cyber-ostracism, depression, experiential avoidance, and non-suicidal self-injury were positively correlated with each other. After controlling for gender and age, the mediation model test shows that cyber-ostracism was significantly and positively associated with non-suicidal self-injury. Depression and experiential avoidance mediated the relationship between cyber-ostracism and non-suicidal self-injury parallelly and sequentially. This study highlights the potential mechanisms of action between cyber-ostracism and adolescent non-suicidal self-injury and finds that cyber-ostracism is a risk factor for non-suicidal self-injury. This founding suggests that extra attention should be paid to the role of the online environment in addition to the offline environment experiences for the intervention of non-suicidal self-injury.

## 1. Introduction

Non-suicidal self-injury (NSSI) is defined as an act that does not contain suicidal intent and does not result in death, where the person intentionally and directly causes injury to themselves, and such an act is not socially acceptable [1]. There are many ways of self-injury, including cutting, scratching, hitting, burning, carving on the body, stabbing with sharp objects, and other deliberate, direct destruction or alteration of body tissue behavior [2]. A review study showed that NSSI is more common in adolescents, with an age of onset between 12 and 14 years and a prevalence of 7.5–46.5% in adolescents [3]. NSSI peaks in adolescence at around 15 to 17 years [4]. Although NSSI is substantially different from suicide, most studies have found NSSI to be a risk factor for suicide. Previous studies found that NSSI increases the risk of transitioning from suicide ideation to attempt, and NSSI is associated with increased odds of subsequent onsets of suicidal thoughts and behaviors [5]. Another study found that 70% of adolescents engaging in recent NSSI reported a lifetime history of at least one suicide attempt [6]. There is no doubt that NSSI has become a topic of concern for researchers all over the world. Given the hazards and prevalence of self-injury, it is important to explore the causes of NSSI and intervene accordingly.

Previous research has found that both individual characteristics (such as personality [7,8], gender [9,10], age [11], and emotion [12,13]) and environmental characteristics (such as school climate [14], family climate [15,16], and peer climate [12,17]) influence adolescents’ NSSI. In terms of environmental factors, several studies have examined the impact on adolescent NSSI in the online context, such as cyberbullying victimization [13,18]. However, in general, there is a relative lack of research that examines the relationship between online environments and NSSI. One study found that the rise of digital media may have some association with mental health; these trends included sharp increases in depression, anxiety, loneliness, self-harm, suicidal ideation, and so on [19]. Based on previous studies, the present study will focus on the variable of cyber-ostracism to further examine the relationship between the online environment and NSSI. It will also explore the possible mediating mechanisms between the two in order to provide reference values for the intervention of adolescents’ NSSI. Specifically, based on the experiential avoidance model, the current study aims to test the relationship between cyber-ostracism and adolescents’ non-suicidal self-injury and to explore the mediating roles of depression and experiential avoidance.

### 1.1. Cyber-Ostracism and Non-Suicidal Self-Injury

Cyber-ostracism is a specific form of social exclusion that occurs on the Internet [20]. With the popularity of the Internet, it is a very common phenomenon for people to socialize through electronic devices. Because of the convenience and breadth of online socialization, as well as its characteristics (such as anonymity, lack of social cues, and transmission rate of media), cyber-ostracism may cause more severe harm to individuals than face-to-face ostracism. Previous research has found that for individuals who are excluded from social media their sense of belonging, self-esteem, meaningful existence, and emotional states were threatened [20,21]. These indicators are closely related to NSSI, so cyber-ostracism may have an association with NSSI. The integrated theoretical model of the development and maintenance of NSSI provides evidence to support this view, which proposes that NSSI has the function of regulating aversive affective experiences and social situations [22]. Cyber-ostracism is often accompanied by negative emotions that are not a pleasant experience. Therefore, individuals may adopt self-injury to change the situation. In addition, the integrated theoretical model suggests that poor interpersonal relationships are also a risk factor for NSSI [22]. Previous studies have found that individuals with poorer peer relationships tend to have more NSSI [12,23]. A longitudinal study also showed that negative interpersonal relationships increased odds of subsequent adolescent NSSI onset [15]. Therefore, we proposed hypothesis 1: cyber-ostracism would be positively associated with NSSI.

### 1.2. The Mediating Role of Depression

As mentioned above, online social rejection may bring some negative emotions. Based on the temporal need-threat model and emotion management model, we believe that depression may play a mediating role between cyber-ostracism and adolescent NSSI. On the one hand, cyber-ostracism may be associated with depression. The temporal need-threat model describes processes at three stages of reactions to ostracism, including reflexive, reflective, and resignation [24]. If ostracism episodes persist over an extended time, individuals may feel alienation, depression, helplessness, and unworthiness during the resignation stage [24]. Previous research has also found that individuals who are excluded by others tend to have higher levels of depression [25,26]. On the other hand, depression may be associated with NSSI. The emotion management model of self-injury suggests that NSSI has an emotion regulation function by changing the current situation, getting out of a painful situation, and distracting from negative emotions or thoughts [27]. Depression is a painful emotion, and in order to escape it, adolescents may resort to self-injury in this way. Previous studies have also found that depressed individuals engage more NSSI [8,12]. Therefore, we proposed hypothesis 2: cyber-ostracism would be positively associated with NSSI through the mediating role of depression.

### 1.3. The Mediating Role of Experiential Avoidance

In addition to depression, experiential avoidance may also mediate the relationship between cyber-ostracism and NSSI. First, cyber-ostracism may be associated with experiential avoidance. Experiential avoidance includes any behavior that functions to avoid or escape unwanted internal experiences or external conditions that elicit them. These avoided experiences may consist of thoughts, feelings, somatic sensations, or other inner experiences that are uncomfortable or distressing [28,29]. Cyber-ostracism is essentially a form of interpersonal rejection, which is an unpleasant experience. Therefore, in order to avoid the unwanted internal experiences triggered by this external experience, individuals may engage in experiential avoidance. Previous research has also found that people experience more avoidance when exposed to negative life events (such as cyber-victimization and parents’ phubbing) [30,31]. Second, experiential avoidance may be associated with NSSI. The experience avoidance model of self-injury assumes that individuals involved in NSSI have a strong ability or tendency to experience avoidance, which is a proximal factor for individuals to develop NSSI [28]. Previous studies have also confirmed that individuals with a high preference for experiential avoidance have more NSSI [32]. Therefore, we proposed hypothesis 3: cyber-ostracism would be positively associated with NSSI through the mediating role of experiential avoidance.

### 1.4. The Multiple Mediating Roles of Depression and Experiential Avoidance

**Figure 1:**
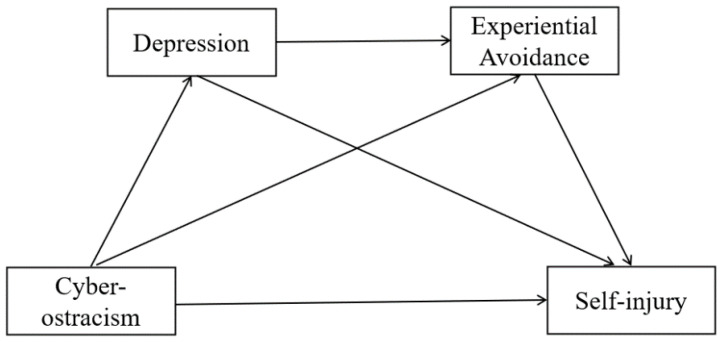
The proposed model.

The experiential avoidance model assumes that the development of negative life stimuli to self-injurious behavior involves two intermediate processes: emotional response and avoidance [28]. Specifically, the occurrence of an irritating event causes the individual to feel negative emotions, which are so distressing that he or she wants to escape. However, due to the inadequacy of emotion regulation strategies, individuals will resort to self-injurious behaviors to quickly relieve the unpleasant experience of negative emotions. The experiential avoidance model of self-injury provides an integrated theoretical framework for the relationship between cyber-ostracism, depression, experiential avoidance, and NSSI. According to the model, cyber-ostracism may be associated with NSSI through the mediating role of depression and experiential avoidance. According to the model, depression may increase the tendency to experience avoidance. Empirical studies have also found a significant correlation between depression and experiential avoidance, in that individuals with high levels of depression have more experiential avoidance [30,33]. In addition, the experiential avoidance model suggests that individuals who are exposed to negative emotions caused by stimuli can temporarily alleviate them by experiencing avoidance. However, this approach negatively reinforces NSSI, creating a vicious cycle. Repeated negative reinforcement strengthens the link between unpleasant emotional arousal and self-injury in this vicious circle. Over time, this leads to NSSI becoming an automatic avoidance response [28]. In summarization, based on the experience avoidance model and related empirical studies, we proposed hypothesis 4: cyber-ostracism would be associated with NSSI through the chain mediating role of depression and experiential avoidance. The hypothetical model is shown in Figure 1.

## 2. Materials and Methods

### 2.1. Participants and Procedure

The present study sampled 1062 middle school students from two junior high schools in the central part of China. Before the questionnaire was distributed, we obtained permission from students, parents, and teachers. The main examiners were trained graduate students majoring in psychology who were familiar with the whole testing process. Participants were asked to answer truthfully and independently, following the principle of voluntariness, and could withdraw at any time if they did not want to do the questionnaire in the process of testing. The mean age of the participants was 13.05 (SD = 0.77, range = 12–16). A total of 503 (47.36%) of the participants were male, and 559 (52.64%) of the participants were female.

### 2.2. Measures

#### 2.2.1. Cyber-Ostracism

The Cyber-ostracism Experience Scale developed by Niu [34] et al. was used. The questionnaire has 14 items and contains three dimensions: cyber-ostracism in online personal chat, cyber-ostracism in online group chat, and cyber-ostracism in personal web space. Examples of items measured include “when I find person online for small talk, I couldn’t get a response”. Response categories ranged from “1 = Never” to “5 = All the Time”. This study used the mean scores across the 14 items, with higher scores indicating a higher frequency of experiencing online social rejection. The scale has good reliability and validity in the Chinese adolescent groups [34]. In this study, Cronbach’s α for the scale was 0.93.

#### 2.2.2. Depression

A revised Chinese version of the depression-anxiety-stress scale (DASS-21) [35] was used. A total of 7 items were scored on a 4-point scale, with “0” indicating “not at all” and “3” indicating “very much”. Examples of items measuring include, “I feel that life is meaningless”. This study used the mean scores across the 7 items, with higher scores indicating a higher level of depression. In this study, Cronbach’s α for the scale was 0.89.

#### 2.2.3. Experiential Avoidance

Bond et al.’s [36] revised Acceptance and Action Questionnaire was used to measure the extent to which individuals avoid internal private experiences they do not want to experience. Seven items were included and scored on a 7-point scale ranging from “strongly disagree” to “strongly agree”. Examples of items measuring include “painful experiences make it difficult for me to live an ideal life”. This study used the mean scores across the 7 items, with higher scores represent higher levels of experiential avoidance. In this study, Cronbach’s α for the scale was 0.92.

#### 2.2.4. Self-Injury

The Adolescent Self-Injury Questionnaire revised by Feng Yu [37] was used. A total of 18 items were used to measure the frequency of self-injury among adolescents. Examples of items measuring include “deliberately scratching own skin with glass, knife, etc.”. The frequency of self-injury was evaluated on four levels: 0 times, 1 time, 2–4 times, and more than 5 times (including 5 times). This study used the mean scores across the 18 items, with higher scores indicating more times of self-injury for the adolescent. In this study, Cronbach’s α for the scale was 0.90.

### 2.3. Data Analysis

First, data screening revealed that there were no outliers in our data. Then, responses with missing data (e.g., gender not reported) were excluded from the data processing. Descriptive analyses were conducted with SPSS to understand the general condition of the variables in this study. Pearson’s correlation analyses were adopted further to examine the potential relationships among all variables. Second, we examined whether our data followed normal distribution before testing our hypotheses. The skewness and kurtosis of adolescents’ depression and experiential avoidance were within the acceptable range (skewness cutoff of 2.0 and kurtosis cutoff of 7.0) [38]. However, cyber-ostracism and NSSI was somewhat positive skewness. We used log transformation on the overall mean scores to approximate normal distributions [39]. The transformed variable was used in our analyses. The mediation model analysis was based on Model 6 proposed by Hayes [40,41] and calculated using Amos. The bias-corrected percentile bootstrap method (with 5000 resamples) was applied to determine whether the effects were significant. The 95% confidence interval does not include 0, indicating a significant mediating effect.

## 3. Results

### 3.1. Preliminary Analyses

Table 1 shows means, SDs, and Pearson correlations for the study variables. As the results showed, cyber-ostracism was positively associated with depression (r = 0.36, *p* < 0.001), experiential avoidance (r = 0.49, *p* < 0.001), and self-injury (r = 0.37, *p* < 0.001). Depression was positively associated with experiential avoidance (r = 0.62, *p* < 0.001) and self-injury (r = 0.51, *p* < 0.001). Experiential avoidance was positively associated with self-injury (r = 0.48, *p* < 0.001).

### 3.2. Testing for the Mediation Model

**Figure 2:**
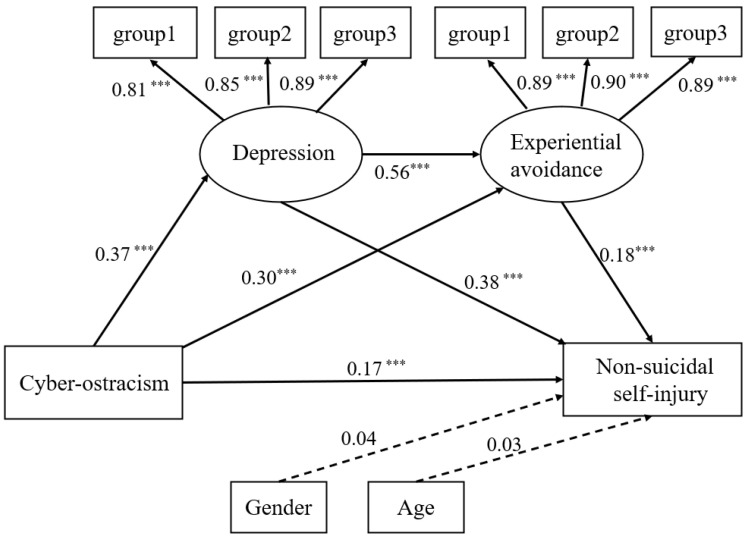
The mediation model. Note: *** *p* < 0.001.

A structural equation model was used to test for mediating effects. Because depression scale and experiential avoidance scale were single-dimensional and had relatively numerous items, we packaged these scales before analysis [42]. Balanced parcels were used [43,44]. The specific steps were as follows. After factor analysis of the questionnaire items, the items were sorted sequentially according to their factor loadings from largest to smallest, then the questions were rotated from high to low and vice versa sequentially according to the number of groups, and finally the items were packaged into observed variables. Specifically, depression was packaged into three observed variables, group 1 (containing items 5, 1, 2), group 2 (containing items 7, 3), and group 3 (containing items 4, 6); experience avoidance was packaged into three observed variables, group 1 (containing items 3, 6, 7), group 2 (containing items 5, 1), and group 3 (containing items 2, 4). The structural equation model was designed with cyber-ostracism as the independent variable, NSSI as the dependent variable, depression and experience avoidance as mediating variables, and gender and age as control variables. Figure 2 shows that cyber-ostracism was significantly and positively associated with depression (B = 0.37, *p* < 0.001), experiential avoidance (B = 0.30, *p* < 0.001), and NSSI (B = 0.17, *p* < 0.001). Depression was significantly and positively associated with experiential avoidance (B = 0.56, *p* < 0.001) and NSSI (B = 0.38, *p* < 0.001). Experiential avoidance was significantly and positively associated with NSSI (B = 0.18, *p* < 0.001). The results show that the mediation model fits well (χ^2^/df = 4.90, GFI = 0.97, AGFI = 0.95, CFI = 0.98, TLI = 0.97, NFI = 0.97, IFI = 0.98, RMSEA = 0.06).

The results of the bootstrap test indicated that depression and experiential avoidance mediated the relationship between cyber-ostracism and NSSI (see Table 2). Specifically, through three pathways, one through the indirect role of depression (indirect effect = 0.14, SE = 0.03, 95%CI = [0.09, 0.20]) accounted for 60.87% of the total indirect effect. One through the indirect role of experiential avoidance (indirect effect = 0.05, SE = 0.02, 95%CI = [0.02, 0.10]) accounted for 21.73% of the total indirect effect. A chain mediating role through depression and experiential avoidance (indirect effect = 0.04, SE = 0.01, 95%CI = [0.02, 0.06]) accounted for 17.40% of the total indirect effect.

## 4. Discussion

In the context of the growing popularity of the Internet, researchers have paid increasing attention to the impact of the Internet on people. However, some researchers have examined the study of the variable of the online environment on adolescent NSSI [18]. However, overall, there are relatively few studies in this area. Based on the experience avoidance model, the present study explored the relationship between cyber-ostracism and adolescent NSSI and the mediating role of depression and experience avoidance. The results showed that cyber-ostracism could be related to adolescent NSSI not only directly but also indirectly through the separate mediation of experience avoidance of depression and the chain mediation of both. The findings further explain why cyber-ostracism is associated with adolescent NSSI and has implications for the intervention of adolescent self-injurious behaviors.

### 4.1. Cyber-Ostracism and Non-Suicidal Self-Injury

The current study explored the relationship between cyber-ostracism and NSSI among adolescents. The results found that adolescents with exposure to cyber-ostracism are more likely to engage in NSSI. That is, cyber-ostracism is a risk factor for adolescents’ NSSI, highlighting the importance of cyber-ostracism in shaping adolescents’ NSSI. This is consistent with previous research findings on the positive association between traditional exclusion and adolescent NSSI [45]. Cyber-ostracism often has painful emotional outcomes, and adolescents may resort to self-injury as a method to alleviate these emotions. The integrated theoretical model of NSSI argues that NSSI has the function of modifying social situations in addition to regulating aversive emotions [22]. Self-injury is a way of communicating with others or influencing them by drawing their attention to him or her. In this sense, self-injury in a social exclusion context may be a call for help from adolescents to the outside world. The findings can also be explained from the self-determination theory perspective, which proposes that people have three innate basic psychological needs for competence, autonomy, and relatedness. When these three basic psychological needs are satisfied, people will develop normally; if the basic psychological needs are hindered, there will be many negative consequences [46]. Cyber-ostracism is a form of interpersonal rejection that can impair individuals’ basic psychological needs and, therefore, may have a harmful effect on adolescents’ mental well-being. Previous research has confirmed that those with poor interpersonal relationships (cyberbullying victimization) can impair adolescents’ autonomy need satisfaction, and further lead to adolescents’ NSSI [18].

### 4.2. The Mediating Role of Depression

As predicted, we found that depression mediates the relationship between cyber-ostracism and NSSI. Specifically, this finding suggests that individuals who experience cyber-ostracism are more likely to have higher levels of depression, which in turn increases NSSI. The hopelessness theory of depression can also explain the relationship between cyber-ostracism and depression. According to this theory, hopelessness is a proximal sufficient cause of the symptoms of hopelessness depression, and expectations of helplessness about changing the likelihood of occurrence of these outcomes (a helplessness expectancy) is one of the main factors contributing to feelings of hopelessness [47]. Individuals who are ostracized by others often lack effective interpersonal strategies, and adolescents may find it difficult to change this outcome of being isolated. As a result, adolescents may be prone to feelings of hopelessness in the face of this event, which may lead to depression. Previous research provides indirect evidence for this inference that individuals who are ostracized develop more depression through an increase in rejection sensitivity and a decrease in interpersonal self-efficacy [25]. Moreover, a longitudinal study also suggested that ostracism could predict depression symptoms among youths [48]. Individuals who are depressed tend to engage in more NSSI behaviors, which is supported by a large body of previous research [12,23]. The findings support the emotion management model that when faced with negative emotions such as depression, individuals may resort to self-injury to regulate their emotions if they lack effective emotion regulation strategies.

### 4.3. The Mediating Role of Experiential Avoidance

This study found that in addition to depression, experiential avoidance also mediated the relationship between cyber-ostracism and adolescent NSSI. The findings further revealed the reasons for the association between cyber-ostracism and adolescent NSSI. The researchers believe that despite its obvious negative consequences, NSSI is quite functional on a certain level, as it may be exceedingly effective at terminating unwanted emotional states [28,49]. The experiential avoidance model considers that NSSI is maintained and strengthened through the process of escape conditioning and powerful negative reinforcement [28]. Essentially, an emotionally evocative event occurs, triggering an aversive emotional response. The individual experiences an urge to escape from the unpleasant state of arousal and engages in NSSI, which reduces or eliminates the emotional arousal. As time goes on, individuals may become more dependent on avoidance and negatively reinforce NSSI, making this behavior considerably more likely when the individual experiences similar conditions in the future.

### 4.4. The Mediating Roles of Depression and Experiential Avoidance

According to the multiple mediation model, cyber-ostracism could also be associated with NSSI through a sequential mediating effect involving depression and experiential avoidance. The results of the study confirmed the research hypothesis. When adolescents are confronted with cyber-ostracism, they develop uncomfortable emotions. Individuals resort to NSSI to obtain instant gratification in order to escape or alleviate the unpleasant emotional experience. The findings confirm the validity of the experiential avoidance model to explain NSSI. NSSI is a motivated behavior based on avoidance of undesirable emotions, and the mechanism of NSSI is inseparable from emotions. Both depression and experiential avoidance contribute to NSSI, and emotional reactions (depression) and experiential avoidance work in sequence. More importantly, this study extended the model’s applicability by including negative events in the online environment (cyber-ostracism) as a distal influence on NSSI.

## 5. Implications and Limitations

The results of this study has some important theoretical and practical implications. From the perspective of theoretical value, the current research enriches the knowledge and literature on the mechanisms connecting the relationship between adverse events (e.g., cyber-ostracism) and NSSI. Furthermore, to some extent, these findings have supported and enriched the experiential avoidance model to explain the adverse effect of the stressor and the mechanism of action. From the perspective of practical value, the findings from the current study suggest that cyber-ostracism is a risk factor for NSSI among adolescents. Therefore, it would be helpful for educational researchers to focus on improving adolescents’ social skills on social networking sites. This will reduce the probability of NSSI through experiencing cyber-ostracism and ultimately reduce the potential risk of NSSI. More importantly, mediation effects suggest that cyber-ostracism can be associated with adolescent NSSI through depression and experiential avoidance. Therefore, they could also be helped through mental health classes, group counselling, and individual counselling to reduce their engagement in NSSI by reducing depression and mastering well-trained emotional coping strategies.

Despite these implications, several limitations should be addressed. First, we draw conclusions based on a cross-sectional method that could not confirm causal relationships. Thus, based on our results, future studies should use longitudinal and experimental designs to explore causal relationships between the variables. Second, this study employed a self-reported questionnaire method, and the results may be influenced by social approval. In the future, more non-intrusive techniques (such as big data analysis) could be used to collect more realistic behavioral indicators from users and obtain more accurate relationships between variables. Third, the present study only explored the mediating mechanisms between negative life events (cyber-ostracism) and self-injurious behavior, and according to the experience avoidance model [28], there are also some moderating variables, such as poor distress tolerance and emotion regulation skill deficit, between the two. Therefore, in future studies, researchers can explore the possible boundary roles between the two in order to further understand how cyber-ostracism is related to NSSI under different conditions. Finally, although the present study controlled for gender and age, previous studies have found that there is a close association between autism and eating disorders and self-injurious behavior [50,51,52,53]. Therefore, both should be included as control variables in future studies concerning self-injury.

## 6. Conclusions

To summarize, this study contributes to the literature by establishing a multiple mediation model, which provides an elaborate understanding of why cyber-ostracism is related to adolescent NSSI. Specifically, we found that cyber-ostracism is a risk factor for increased NSSI. Moreover, cyber-ostracism increases the risk of NSSI via the parallel mediating effects of depression and experiential avoidance, as well as via the sequential mediating effects of depression and experiential avoidance.

## Figures and Tables

**Table 1:** Descriptive statistics and correlations for the main variables.

Variable	M	SD	Skewness	Kurtosis	1	2	3	4
1. Cyber-ostracism	0.16	0.15	1.09	0.69	—			
2. Depression	0.49	0.66	1.66	3.97	0.36 ***	—		
3. Experiential Avoidance	2.41	1.47	1.20	0.77	0.49 ***	0.62 ***	—	
4. NSSI	0.07	0.10	2.01	2.63	0.37 ***	0.51 ***	0.48 ***	—

Note: **M** is short for the mean, **SD** is short for the standard deviation; NSSI is short for non-suicidal self-injury; ***N*** = 1062, *** *p* < 0.001.

**Table 2:** Bootstrap test of path.

Path	Standard Estimate	S.E.	[Boot 95% CI]
Indirect_All	0.23	0.03	[0.18, 0.29]
Cyber-ostracism → Depression → NSSI	0.14	0.03	[0.09, 0.20]
Cyber-ostracism → Experiential Avoidance → NSSI	0.05	0.02	[0.02, 0.10]
Cyber-ostracism → Depression → Experiential Avoidance → NSSI	0.04	0.01	[0.02, 0.06]

## Data Availability

To protect the participants’ privacy, the original data used for the analysis are not publicly available but from the corresponding author at a reasonable request.
